# The tuberous sclerosis complex gets fatter

**DOI:** 10.18632/oncotarget.18436

**Published:** 2017-06-11

**Authors:** Prashanth Gokare, Wafik S. El-Deiry

**Affiliations:** Department of Hematology/Oncology and Molecular Therapeutics Program, Laboratory of Translational Oncology and Experimental Cancer Therapeutics, Fox Chase Cancer Center, Philadelphia, PA, USA

**Keywords:** TSC, cholesterol, chloroquine, mTOR, endosomes, lysosomes

Tuberous sclerosis complex (TSC) is a pleomorphic disease affecting multiple organs such as the brain, kidney, lungs, skin, eyes etc [1]. It is characterized by germline loss-of-function mutations in TSC1 and TSC2 genes or inactivation of the TSC complex. mTORC1 pathway activation plays a central role in the pathophysiology associated with TSC. Even though the complete molecular underpinning of various manifestations of TSC are not yet fully understood, the recent report from Elizabeth Henske and colleagues (Filippakis *et al*. [2]) highlights a novel aspect of TSC biology. Since mTORC1 is usually found associated with the lysosome, its contribution to TSC pathophysiology to some extent till now has remained unknown. In the current study the authors found out that TSC-deficient cells, which have hyperactive mTORC1 activity, show an enriched gene expression signature belonging to the cholesterol biosynthesis pathway upon inhibition of lysosome-autophagosome formation by chloroquine (CQ). CQ treatment further enhanced total and esterified cholesterol accumulation in lysosomes of Tsc2^−/−^ cells compared to Tsc2^+/+^ (and to a lesser extent free cholesterol). To analyze the contribution of the *de-novo* synthesis of cholesterol to the overall increase in cholesterol following CQ treatment, simvastatin, an HMG-CoA reductase inhibitor (which blocks *de-novo* cholesterol synthesis) was used in combination with CQ. Surprisingly co-treatment with CQ strikingly rescued the cell death effects of simvastatin. Genes responsible for both cholesterol synthesis and uptake were upregulated following the combination drug treatment. However, the rescue of the cell death phenotype was primarily dependent upon the marked uptake of exogenous lipids via LDLR upregulation. The uptake of exogenous lipids was mTORC1-dependent and SRBEP2 played a central role in the upregulation of genes involved in cholesterol homeostasis including LDLR. Perhaps one of the key observations made by Henske and colleagues was that, the blocking of NPC1 which pumps out free cholesterol from the lysosome phenocopied CQ effects, suggesting that lysosomal accumulation of cholesterol-esters contribute yet through an unknown mechanism towards the survival of *de-novo* and lysosome-inhibited cholesterol biosynthesis. An important mediator of this effect was identified as Vps34, a critical protein kinase required for endosomal lysosomal trafficking. Pharmacological inhibition of Vps34 combined with CQ significantly reduced the cell survival effects mediated by CQ. Further genetic targeting of LDLR completely abrogated the survival of simvastatin and CQ treated TSC-null cells reiterating that the exogenous uptake of cholesterol is a crucial factor for cell survival under lysosomal and *de novo* cholesterol synthesis inhibition.

Currently TSC management depends on the local manifestations of the disease, clinical features, early screening, diagnosis and surgical removal in some cases. Promising results in clinical trials for pulmonary Lymphangioleiomyomatosis, Subependymal Giant Cell Astrocytoma (SEGA) and Renal angiomyololipoma (AML), which are major manifestations of TSC, with mTOR inhibitors have recently led to FDA and EDA approval of these agents. Novel drugs targeting metabolic genes have emerged providing encouraging results in early preclinical models. For an extensive review on the current state of clinical management of TSC please refer to Henske *et al* [3]. The study highlighted here has important implications towards the current understanding of TSC pathogenesis and treatment and also raises important questions. First, how does cholesterol accumulation in the lysosome benefit the survival of TSC-null cells. It would be of interest to delineate specific pathways that might depend on cholesterol availability in the lysosomes to promote cell survival. A recent report by Castellano *et al*. [4], identified that lysosomal accumulation of cholesterol can activate mTORC1 through interaction between SLC38A9 and NPC1. Knowledge of these underlying pathways might pave the way for exploring synthetic lethal interactions and combination treatments with mTOR targeting. Second, the extent to which the results are generalizable including the dependency on increased cholesterol synthesis is of interest for the TSC spectrum disorders. In support of the current findings a recent study has observed an increased serum lipid profile in SEGA patients who have undergone everolimus therapy for 12 months [5]. Future work can unravel whether the mechanistic underpinnings revealed may recapitulate in mouse models of TSC. The current study by Filippakis *et al*. provides novel insights into cholesterol homeostasis, endosomal and lysosomal interactions that further extend our knowledge towards the continued quest for understanding and treating TSC and related disorders.
